# Recovery of Muscular Strength Following Total Hip Replacement: A Narrative Review

**DOI:** 10.7759/cureus.68033

**Published:** 2024-08-28

**Authors:** Gursimran Singh, Nareshkumar Dhaniwala, Vivek H Jadawala, Anmol Suneja, Nitish Batra

**Affiliations:** 1 Orthopaedics, Jawaharlal Nehru Medical College, Datta Meghe Institute of Higher Education and Research, Wardha, IND; 2 Medicine, Jawaharlal Nehru Medical College, Datta Meghe Institute of Higher Education and Research, Wardha, IND

**Keywords:** physical therapy, hip surgery, functional recovery, strength training, rehabilitation, postoperative recovery, total hip replacement, muscular strength

## Abstract

This narrative review analyzes muscle strength recovery following total hip replacement (THR) and looks at various factors affecting postoperative muscle function restoration. The review synthesizes evidence from various studies regarding the timing and degree of muscular strength recovery, different rehabilitation protocols, and patient-specific variables such as age, preoperative physical condition, and comorbidities, among others. Overall, it appears that THR is associated with improved hip function and quality of life, but this usually takes a long time due to individualized physical therapy interventions. In addition, postoperative rehabilitation has been found not to exist without any personal factors involved such as age or gender whereby for instance senior citizens have no alternative but to go for THR surgery, making their lower limbs weaker than those who are younger. Based on the findings in this review on muscle recovery after THR surgeries, one may conclude that this endeavor should begin as early as possible and include regular resistance training programs with performance-focused functional training after surgery. In addition, more longitudinal studies should be conducted regarding post-surgical outcomes comparing other traditional medical practices.

## Introduction and background

Total hip replacement (THR) is a surgical procedure that may provide pain relief and restore functions, particularly to individuals with severe cases of hip arthritis or other conditions that hinder the normal functioning of their hips. Postoperative rehabilitation focused on THR must involve recovery of muscular strength because this contributes to the best functional outcomes and improved quality of life [1, 2]. Recovery after THR mainly depends on hip joint muscle power, which determines walking movements, balance, and daily activities such as walking a short distance. The loss in muscular strength occurs due to both the underlying condition requiring THR and the surgery itself. Such a decrease may impede recovery time or completeness, thus extending regrettable long-term disability or low satisfaction among patients [3]. Furthermore, insufficient muscular force can slow healing after surgery, resulting in abnormal gait patterns and impaired mobility [4]. Recovery from THR has been studied by various researchers who documented numerous factors influencing the rehabilitation process such as pre-surgery muscle fitness, surgical modalities, postoperative rehabilitation protocols, and the use of exercise programs by patients [5, 6]. This narrative review aims to consolidate existing research on the recovery of muscular strength following THR. By examining the current evidence, this review seeks to identify critical factors affecting recovery, evaluate the effectiveness of different rehabilitation strategies, and provide recommendations for improving patient outcomes.

## Review

Search methodology

A detailed search strategy was used to perform a narrative review regarding the recovery of muscular strength after total hip arthroplasty (THA). We systematically scoured electronic databases, such as PubMed, Embase, and Cochrane Library, for relevant articles published until August 2024. Search terms included various phrases indicating “muscular strength recovery,” “total hip replacement,” and “postoperative rehabilitation.” We incorporated studies that concentrated on the return of muscular strength among adults following THR, with results assessed using strength evaluations, functionality tests, or related measures. Articles were required to be peer-reviewed with an English-language publication. Excluded were those related to non-muscular recovery, animal studies, and those that did not have clear indicators specifically about muscle power enhancement. Also excluded were reviews, editorials, or research targeting non-specific populations or subjects irrelevant to the study. This method enabled comprehensive and focused analysis of the evidence surrounding muscle power recovery after THR surgery.

Muscular strength recovery post-total hip replacement

Impact of Total Hip Replacement on Muscle Function and Strength

THR is one of the most frequently performed surgeries for treating severe hip joint arthritis and other hip diseases. In this procedure, the damaged hip joint is replaced by a prosthesis, which can significantly impact muscle functioning and strength.

Muscle Function and Strength After Total Hip Replacement

Postoperative muscle function and strength are important determinants of recovery and overall functional outcomes. The surgical operation usually leads to significant muscle destruction, particularly affecting hip abductors, flexors, and extensors. Post-THR muscle weakness may occur as a result of direct trauma during surgery and later disuse of affected muscles during recovery. This muscular weakness may impair walking or performing activities that require moving from one place to another, leading to an increased risk of falling and decreased life quality [7]. Literature shows that muscle strength deficits usually occur immediately after the operation, which can persist if not addressed early with rehabilitation [8]. Such deficits might delay the return to physical functioning and influence long-term results such as frequency of physical exercises or mobility level [9].

Role of surgical and rehabilitation factors

The functional performance of muscles and recuperation after the operation depends highly on the surgical method used in THR. Compared with conventional open methods, minimal invasiveness in surgery has been proven to mitigate muscle injury and accelerate recovery [10]. For instance, Gao et al. stated that patients subject to minimally invasive THR developed less muscle damage while regaining strength quicker than those who underwent radical hip replacement techniques [11].

The optimization of muscle recovery and functionality after THR requires effective rehabilitation. Therefore, a well-structured program combining passive and active exercises can improve a person’s muscle strength, promote movement, and enhance functional locomotion skills. There are several benefits of early postoperative rehabilitation focusing on strengthening exercises (strength) for improved muscle strength, functional activities, and overall recovery outcomes [12]. In addition, it has been shown previously that incorporating strength training exercises targeting hip abductors and extensors appears to be particularly beneficial in addressing post-THR muscle weakness [13]. Finally, the timing and intensity of rehabilitation interventions also significantly impact patient consultative capacities. A vital part of avoiding muscle atrophy is doing progressive resistance training during the initial stages of mobilization. Meier et al. (2008) found that patients who engaged in early postoperative rehabilitation experienced faster improvements in muscle strength and functional mobility than those with delayed rehabilitation protocols [14].

Factors influencing recovery

Preoperative Muscle Strength and Conditioning

Preoperative muscle strength and conditioning make up an important part of the recovery process after THR. Improved postoperative outcomes due to enhanced muscle function and overall physical fitness results from preoperative physical conditioning, including strength training and aerobic exercises [15]. Faster recovery and fewer complications are often reported by patients with good muscle strength before surgery. Muscle wasting and weakening that often occur in patients undergoing THR can be prevented through preoperative strength training that mostly targets the hip, quadriceps, and core muscles [16].

Surgical Technique and Implant Type

The surgical method and implant used can have a considerable effect on regaining muscle power after THR. Techniques that involve less cutting, such as those involving minimal access to the body parts involved, have been seen to lead to reduced trauma to the body tissues, hence fewer recovery times compared to conventional surgeries [17]. The type of implant, including its material and design (for instance, whether it is cemented or cementless), also affects how much strength and movement function is regained by muscles after an operation [18].

Postoperative Rehabilitation and Exercise Programs

After THR, rehabilitation and exercise programs are essential to the recovery of muscular strength. To restore muscle strength and joint function, structured rehabilitation programs that include progressive resistance training, functional exercises, and gait training are necessary [19]. The initial mobilization and strict compliance with rehabilitation protocols have been linked to improving outcomes such as muscle strength, range of motion (ROM), and functional performance [10]. Moreover, exercise programs that align with the personal needs of individuals at different stages of recovery can greatly affect the rate and degree of recovery [20].

Rehabilitation programs

Rehabilitation following THR is crucial for restoring muscular strength, joint function, and overall mobility. Various exercise types are incorporated into rehabilitation programs, each with specific benefits and effectiveness:

Range of Motion Exercises

ROM activities can improve flexibility and joint movement. Stretching, rotating, flexing, and extending the knee are common movements for a joint’s ROM exercise. Using ROM exercises at an early stage has been shown to significantly enhance joint mobility and decrease pain levels. ROM serves as a basic unit in physical therapy and rehabilitation programs aimed at improving or maintaining the ROM in joints. They entail moving a joint through all its available arcs of motion in every direction that it would normally allow. For this reason, various forms of ROM exercises help keep joints healthy, prevent stiffness, and facilitate muscle relaxation and other functions involved with pliability [21].

Strengthening Exercises

Strengthening exercises focus on rebuilding the strength of muscles around the hip joint. Key exercises include isometric, resistance, functional, and aerobic exercises.

Isometric exercises involve contracting the muscles without changing the joint angle, such as isometric hip abduction and adduction. They help maintain muscle strength while minimizing joint stress. THR follows a recovery routine that relies heavily on isometric exercises. These exercises involve tightening muscles without moving the joint, making them especially useful during the early stages of recovery when the joint is still mending and mobility is restricted. Isometric exercises are pivotal in sustaining and gradually regaining muscle power around the hip joint while avoiding putting too much strain on the operated area [22].

Resistance training includes exercises using resistance bands or weights to strengthen the hip flexors, extensors, and abductors. Research shows that resistance training significantly improves muscle strength and functional outcomes post-THR [23].

Functional exercises mimic daily activities including squats, step-ups, and balance exercises. Several methods can be employed in rehabilitating individuals after undergoing THR surgery. One method that is often used is the performance of functional exercises. These workout routines aim to help patients regain their strength, mobilize their joints, and develop stamina so they can go back to doing things on their own and having an active way of life again. In essence, these workouts increase how the whole joint works and its neighboring muscles, thereby enhancing recovery [24].

Low-impact aerobic exercises, such as walking, cycling, and swimming, are included to improve cardiovascular fitness and endurance. Evidence suggests that aerobic exercises support overall recovery and functional improvement. When it comes to taking care of oneself, aerobic exercise is an essential part of the rehabilitation period after THR. Thus, these exercises are intended to stimulate the heart and lung system, build stamina, and boost bodily health. It is important for both healing and sustaining surgical achievement. Adding aerobic workouts to the recovery plan will greatly affect how well the patient moves around, functions, and lives compared to if these workouts were not included [25].

A combination of these exercises has been shown to improve muscle strength, reduce pain, and enhance overall function following THR. Studies consistently report better recovery outcomes with comprehensive rehabilitation programs incorporating varied exercise types [26].

Frequency, intensity, and duration of rehabilitation

The frequency of rehabilitation sessions is influenced by the patient’s condition and the stage of recovery. The general recommendation is to begin by performing rehabilitation exercises 2-3 times a week initially before increasing gradually as recovery progress is made [23]. The acute recovery phase right after surgery may require more sessions, while later stages would call for fewer but intensive sessions.

Adapting rehab movements according to the patient's specific needs is a must since they experience different pain levels. For instance, experts recommend low exercise intensities during the early stages of surgical healing to avoid overstraining recovering body parts. However, these workouts may grow progressively more vigorous for bettering muscles and sustaining endurance over time. Tracking and adjusting your exercises rightly means no harm can come to you while achieving maximum results [22].

The duration of rehabilitation programs varies but typically spans several weeks to months. Initial rehabilitation may focus on shorter, more frequent sessions, gradually transitioning to longer and more intensive sessions as strength and function improve. Comprehensive rehabilitation programs often last 8-12 weeks, with continued home exercise recommendations beyond this period to maintain and further improve stability and function [24].

Outcomes and measures

In the context of recovery following THR, several metrics are frequently employed to assess muscle strength. Commonly used metrics for assessing muscle strength can be broadly categorized into isometric strength tests and functional assessments.

Isometric Strength Tests

Isometric strength tests represent a major aspect in understanding muscular function and the recovery process following THR. They quantify force exerted without visible movement at the affected joint, thus measuring precise control of muscle strength. In THR, isometric strength tests become more meaningful when clinicians use them to examine healing in the hip joint and its adjoining muscles because they play an important role in helping people regain their mobility while preventing problems such as joint instability or walking abnormalities. Commonly used isometric strength tests include isometric hip abduction and isometric quadriceps strength tests.

The isometric hip abduction test measures the strength of the hip abductors, which are crucial for stabilizing the pelvis and maintaining proper gait. The isometric hip abduction test is an important clinical tool to assess the strength and functionality of the hip abductor muscles following THR. It involves maximal voluntary contraction of the hip abductors while lying on the affected side, with the therapist placing the hands above and below it. To perform this test, ask the patient to lift their leg laterally against resistance and maintain contraction without moving the hip joint. Evaluation of gluteus medius and minimus is crucial because they play a significant role in stabilizing the pelvis during walking. Losing strength in these muscles can change gait patterns, reduce mobility, and escalate the risk of falls after THR. Hence, the isometric hip abduction test should be conducted promptly postoperation. This assessment guides individualized rehabilitation aimed at strengthening muscle functions and enhancing the quality of life due to improved mobility after THR surgeries [27].

The strength of the quadriceps muscle is measured by asking the patient to extend the knee against resistance while seated. The isometric quadriceps strength test is a pivotal evaluation tool in assessing patients who have undergone THR to determine functional recovery of the quadriceps muscle. This test involves maximizing voluntary quadriceps contraction without joint movement for the patient against a fixed resistance, enabling reproducible and controlled muscle strength measurement. The quadriceps muscles play an important role in knee stabilization. Hence, its strength becomes important for postoperative recovery and overall lower limb functionality. Such tests help assess how effective rehabilitation modalities are, guide modification of physical therapy regimes, and even project regaining independence in daily living activities by the patient. Post-THR quadriceps strength deficits detected through these assessments underline targeted strengthening exercises in rehabilitation plans so that they can enhance recovery and promote better outcomes over time [10].

Functional Assessments

Functional assessments evaluate muscle strength in the context of performing everyday activities. These assessments often correlate more directly with the patient’s ability to perform daily tasks and regain functional mobility. Critical functional assessments include the timed up and go (TUG) test, the six-minute walk test (6MWT), and the gait speed test.

TUG test represents a notable instrument for assessing patient performance, specifically those recovering from THR. For this trial, the time taken by the patient to rise from the chair, walk three meters, turn around, return to the chair, and then sit down is recorded. This is a quick way to determine how well someone can do important things such as walking or standing up. It is particularly useful in the case of THR as it can be used to assess improvements in gait speed (the rate at which an individual walks), balance, and postoperative recovery while identifying patients at risk of falling or those needing additional rehabilitation. A shorter TUG time after surgery indicates good functional outcomes and successful rehabilitation; thus making it an indispensable instrument for monitoring recovery progress after successful surgery and managing postoperative care strategies (including physical therapy) [28].

6MWT gauges the distance a person can travel on foot within six minutes. It provides an objective assessment of aerobic capacity and endurance, both essential indicators for recovery following THR surgery. Specifically, the 6MWT is valuable because it reflects the patient’s ability to carry out daily activities, thereby serving as an indicator of global functional mobility. Consequently, post-THR improvement in 6MWT distance is typically used to determine whether rehabilitation programs have achieved their intended aim, with longer distances implying better restoration of physical function. Moreover, the test is easy to conduct, requires no special tools, and is therefore acceptable by patients, thus making it suitable for monitoring progress during the postoperative phase. Furthermore, the 6MWT may also help identify individuals who require additional interventions, aimed at optimizing recovery and promoting personalized rehabilitation programs [29].

The gait speed assessment is a significant instrument for evaluating functional mobility and recovery in patients after THR. During this test, a patient walks a fixed distance at their usual pace, usually 10 meters, and the time taken is measured. It gives objective walking speed data essential in determining total functional capacity and lower limb strength. In terms of THR, gait speed can serve as an index of the effectiveness of rehabilitation measures and how the surgery affects moving around. Also, faster movement has been associated with better recovery outcomes; therefore slowness may be seen as indicating difficulties with movement or persisting pain. Clinicians use gait speed routinely after surgery to help them tailor their rehabilitation approaches and notice changes that occur in rehabilitation strategies with time leading to a more efficient personalized recovery plan [30].

Variability in recovery outcomes across studies

Study Design and Methodology

Variations in study design, including differences in sample size, intervention protocols, and follow-up periods, contribute to inconsistent outcomes. Some studies may use different rehabilitation protocols that can lead to variability in muscle strength recovery [31].

Measurement Techniques

Differences in the metrics and methods used to assess muscle strength can lead to variability. For example, some studies may use isometric tests, while others rely on functional assessments or a combination. The choice of measurement technique can affect the reported outcomes [32].

Patient Characteristics

Individual patient factors can influence recovery outcomes. These include age, preoperative muscle strength, comorbidities, and adherence to rehabilitation programs. Older patients or those with pre-existing muscle weakness may experience slower recovery [33].

Rehabilitation Protocols

Variability in rehabilitation protocols, including the type, intensity, and duration of physical therapy, can lead to differences in recovery outcomes. Studies employing different approaches to rehabilitation may report varying degrees of muscle strength recovery [23].

Outcome Reporting

The inconsistency in outcome reporting, including the lack of standardized measures and variations in reporting methods, contributes to variability in recovery outcomes. Some studies may focus on short-term outcomes, while others may assess long-term recovery, leading to differences in reported results [34].

Clinical implications

Guidelines for Rehabilitation

Early mobilization: Initiate rehabilitation exercises as soon as possible after surgery to enhance muscle recovery and functional outcomes. Early mobilization has been shown to reduce hospital stay and improve postoperative strength and function [35].

Progressive resistance training: Incorporate progressive resistance exercises to address muscle weakness and imbalances. Exercises targeting hip abductors, extensors, and flexors are particularly beneficial in regaining strength [36].

Functional training: Emphasize functional training that mimics daily activities to improve practical outcomes and overall quality of life. Functional tasks, such as stair climbing and walking, should be included in the rehabilitation program [37].

Tailored programs: Customize rehabilitation programs based on individual patient needs accounting for preoperative muscle strength, comorbidities, and surgical factors. Personalized programs have been shown to enhance recovery and muscle strength [38].

Ongoing assessment: Regularly assess and adjust the rehabilitation program to address progress and emerging issues. Continuous monitoring and adaptation are essential for optimizing recovery [23].

Patient Education

Understanding rehabilitation goals: Educate patients about the importance of rehabilitation in recovering muscular strength and improving functional outcomes. Clear communication of goals helps set realistic expectations and foster adherence [39].

Self-management techniques: Teach patients self-management techniques, including proper exercise techniques, home exercise programs, and strategies for managing postoperative pain and swelling. Empowering patients with knowledge enhances their engagement and recovery [40].

Pain management: Guide patients in managing pain and discomfort during rehabilitation. Effective pain management strategies are critical for encouraging participation in rehabilitation exercises [41].

Lifestyle modifications: Advise patients on lifestyle modifications that support recovery, such as maintaining a healthy diet, managing weight, and avoiding activities that may jeopardize the surgical outcome [42].

Multidisciplinary Approach

Coordination of care: Collaborate among orthopedic surgeons, physiotherapists, occupational therapists, and other healthcare professionals to create and implement a comprehensive rehabilitation plan. Coordinated care ensures that all aspects of recovery are addressed [43].

Integrated therapies: Integrate various therapeutic modalities, such as manual therapy, aquatic therapy, and neuromuscular re-education, to address different facets of recovery. A combination of therapies can enhance overall outcomes [31].

Regular communication: Foster regular communication among team members to monitor patient progress, address challenges, and adjust treatment plans as needed. Effective communication helps in delivering cohesive and patient-centered care [44].

Patient-centered care: Ensure that patient is actively involved in their rehabilitation process and that their preferences and concerns are considered. Patient-centered care enhances adherence to the rehabilitation program and improves outcomes [45].

Future directions and research needs

Future research on the recovery of muscular strength following THR should focus on longitudinal studies that track strength gains over extended periods and identify factors influencing long-term recovery. Investigations should explore the impact of various rehabilitation protocols, including resistance training, proprioceptive exercises, and personalized physiotherapy regimens on strength outcomes. Additionally, research should assess the role of preoperative muscle conditioning and postoperative nutritional support in enhancing recovery. Exploring the genetic and biological factors contributing to variability in muscular strength recovery can provide insights into personalized treatment approaches. Lastly, there is a need for studies that evaluate the effectiveness of integrating digital health technologies, such as wearable sensors and telerehabilitation platforms, to monitor progress and tailor interventions in real time.

## Conclusions

In conclusion, the recovery of muscular strength following THR is a critical factor influencing overall rehabilitation and functional outcomes. This narrative review highlights that while patients generally experience significant improvements in muscular strength after surgery, the extent and speed of recovery are influenced by various factors, including preoperative muscle strength, adherence to postoperative rehabilitation, and individual patient characteristics. Early and consistent engagement in targeted physical therapy, including resistance and functional exercises, is essential for optimizing strength recovery and enhancing mobility. Future research should continue to explore individualized rehabilitation strategies and the long-term effects of different therapeutic approaches to further improve recovery outcomes and quality of life for THR patients.
